# Fear of hypoglycemia and quality of life in young people with type 1 diabetes and their parents in the era of sensor glucose monitoring

**DOI:** 10.3389/fendo.2022.958671

**Published:** 2022-07-28

**Authors:** Vivien Glocker, Sara Bachmann, Melanie Hess, Gabor Szinnai, Marie-Anne Burckhardt

**Affiliations:** ^1^ Pediatric Endocrinology and Diabetology, University Children’s Hospital Basel, and Department of Clinical Research, University of Basel, Basel, Switzerland; ^2^ Medical School, University of Basel, Basel, Switzerland

**Keywords:** type 1 diabetes, pediatric, intermittently scanned continuous glucose monitoring (isCGM), continuous glucose monitoring (CGM), parents, fear of hypoglycemia, quality of life

## Abstract

**Introduction:**

It is crucial to understand psychosocial outcomes in children and adolescents with type 1 diabetes (T1D) and their families to provide optimal family-centered care. Hence, the aim of this study was to explore psychosocial outcomes in young people with T1D and their parents using currently available glucose monitoring devices in a real-life clinic setting.

**Methods:**

Children and adolescents aged 2-18 years with T1D for more than 6 months and their parents were recruited into a cross-sectional study to complete the Hypoglycemia Fear Survey (HFS) and the Pediatric Quality of Life Inventory (PedsQL) Generic Score Scales, Diabetes Module and Family Impact Module. Demographics and diabetes-specific parameters were obtained from medicals records.

**Results:**

Fifty-nine children and adolescents (mean age 15.1 ± 3.0 years) and 49 parents of children (mean age of children 12.5± 3.3 years) of which 44 were child-parent dyads completed the questionnaires. Parents had a higher mean (SD) FOH total and worry subscore than children, total score was 37.9 (14.6) vs. 32.2 (11.9), p = 0.047 and worry subscore was 17.8 (10.4) vs. 12.8 (9.0), p = 0.01. Furthermore, lower parental diabetes-specific QoL score was observed in parents, 78.8 (12.2) vs. 82.7 (10.3), p=0.02. No difference in FOH and QoL between real-time continuous glucose monitoring (rtCGM) and intermittently scanned glucose monitoring (isCGM) users and users of devices with and without alerts was observed. In isCGM users (n=36 completing the child questionnaires, n=33 completing parent questionnaires), higher parental FOH and lower parental diabetes-specific QoL correlated with higher scanning frequency, r = 0.399, p = 0.021, and r = -0.464, p = 0.007 respectively. No significant correlation was documented between scanning frequency and child questionnaire scores.

**Conclusions:**

Parents are more likely to perceive higher levels of psychosocial burden related to their child’s diabetes than children and adolescents with T1D, especially parents of younger children. This highlights the need for family-based education and treatment resources to support parents in diabetes management in addition to rapidly advancing diabetes technology. In isCGM users, higher parental FOH and lower parent-perceived QoL correlated with a higher scanning frequency, indicating the potential impact of glucose monitoring modality on psychosocial outcomes or vice versa.

## Introduction

Despite significant advances in diabetes technology during the last decade, the majority of children and adolescents with type 1 diabetes (T1D) do not achieve the recommended glycemic targets (1, 2). Suboptimal glucose control is not only associated with an increased risk for acute and long-term complications but also affects health-related quality of life (QoL) (3, 4). Suboptimal glycemic control may influence psychosocial coping of children with T1D and their parents which in turn may affect glycemic outcomes. Daily self-management and the support of family members have a major impact on biopsychosocial outcomes in young people with T1D (5, 6). Daily diabetes management is complex for children and their caregivers and can lead to stress in both children and adolescents. Pre-existing suboptimal glycemic control in youth is often a result of multiple influencing factors, one of these factors can be suboptimal treatment adherence (7). This can lead to family conflicts that can deteriorate suboptimal glycemic control (8). Among others, fear of hypoglycemia even without documented low glucose levels seems to be a major determinant in increasing the psychological burden for children with T1D (9). Moreover, hypoglycemia avoidance behavior due to parental FOH can negatively affect glycemic control of their children (10). Parents often report fear of nocturnal hypoglycemia to be a major problem (11). Thus, to decrease parental anxiety and deducible behavior, interventions, such as optimized diabetes education and diabetes technology, may be helpful to break this potentially vicious circle (10).

The conventional blood glucose monitoring method known as self-monitoring of blood glucose (SMBG) is being progressively enhanced and increasingly replaced with intermittently scanned continuous glucose monitoring (isCGM) and real-time continuous glucose monitoring (rtCGM) in everyday clinical practice and has the potential to improve HbA1c among young people with T1D aged 1-25 years (12, 13). Access to isCGM and rtCGM determines utilization and depends on economic costs (14). In Switzerland, isCGM is less expensive than rtCGM, and is currently covered by health insurances, reimbursement for CGM is granted under certain conditions (15). Hence the majority of young people are using either isCGM closely followed by rtCGM to monitor glucose excursions. This increased uptake of isCGM and rtCGM, and subsequent reduction of having to prick fingers may decrease the burden of diabetes management.

Randomized controlled studies evaluating psychosocial outcomes related to CGM showed that rtCGM and isCGM have the potential of reducing FOH and improving quality of life in children and adolescents and their parents (16, 17). Furthermore, it has been shown that FOH was reduced in children and their parents following a nationwide rtCGM subsidy in Australia (18). An ancillary study of the JDRF CGM Study Group trial reported higher FOH and lower QoL scores in parents of children with T1D compared to the child scores (19). However other studies did not find a difference between children and their parents and the impact of CGM use on child vs. parent FOH is still debated in literature (20). As rtCGM and isCGM are increasingly integrated in the standard of care, a better understanding assessing patient-reported outcomes including psychosocial factors such as FOH and QoL in children and adolescents with T1D and their families in everyday life is crucial. Hence, the purpose of this study was to explore FOH and QoL in children and adolescents with T1D and their parents in the context of currently used glucose sensors in an everyday diabetes clinic setting.

## Methods

### Study design

This prospective exploratory cross-sectional study was conducted at a single tertiary teaching children’s hospital in the German-speaking part of Switzerland between February 2020 and January 2021. After checking eligibility and having consented to the study, demographic data was collected from the medical records. The children and adolescents and their parents were asked to complete validated questionnaires regarding psychosocial factors such as FOH and quality of life.

### Study participants

Children and adolescents with T1D were recruited from the population of patients regularly attending the Diabetes outpatient clinic at the University Children’s Hospital Basel (UKBB). Inclusion criteria were age below 20 years, a diabetes duration greater than 6 months, using multiple daily injections (MDI) or an insulin pump [continuous subcutaneous insulin infusion (CSII)] and giving written informed consent. Children and adolescents and their parents with insufficient knowledge of project language were excluded. The study was approved by the local ethics committee (EKNZ 2019-02462).

### Study protocol

On the occasion of a regular clinic visit, children and their parents were informed about the study and invited to participate. After providing written informed consent by the children and/or parents/guardians, participants (older than 8 years) and one parent (primary caregiver/legal guardian) were asked to complete the validated questionnaires comprising the Hypoglycemia Fear Survey (HFS) and three Pediatric Quality of Life Inventory (PedsQL) modules: the PedsQL 4.0 Generic Core Scales, the PedsQL 3.2 Diabetes Module (21), and the PedsQL 2.0 Family Impact Module (22). Demographic and diabetes management data such as age, gender, diabetes duration, weight, HbA1c, insulin regimen, insulin dose and type of glucose monitoring device (SMBG, isCGM without or with alerts or CGM) were collected. Furthermore, glucose data from the past 14 days were downloaded from their glucose monitoring device.

### Outcome measures

The main outcomes of the study were fear of hypoglycemia and quality of life in children with T1D and their parents. The HFS (child and parent version) consists of 25 items measuring behaviors to prevent hypoglycemia and worry about experiencing a hypoglycemic episode. Every item is scored on a 5-point Likert scale. The HFS calculates a total fear score (possible range 0-100), with subscale scores for behavior that measures behaviors related to preventing an episode of hypoglycemia (range 0-60), and for worry, that measures worry about experiencing a hypoglycemic episode and worry (range 0-40). Higher scores indicate greater FOH. Internal consistency of the HFS was similar to consistency levels reported previously (23): Alpha levels for the respective scales were: Child-HFS total score 0.81, Child-HFS behavior 0.64, Child-HFS 0.85, and Parent-HFS total score 0.90, Parent-HFS behavior score 0.78, Parent-HFS worry score 0.89.

The Pediatric Quality of Life Inventory (PedsQL) Generic Score Scales used in this study comprised of the 23-item PedsQL 4.0 Generic Core Scales, the 28-item PedsQL 3.2 Diabetes Module (21) and the 36-item PedsQL 2.0 Family Impact Module with two subscales (Parent health-related quality of life (HRQL) and family functioning) (22). All parents completed the parent proxy version of the Generic Core, Diabetes Module and the 36-item Family Impact Module. Children aged eight years and older completed the self-report of the Generic Core and Diabetes Module. Each item of the PedsQL Inventory is scored on a 5-point Likert scale to give a total score between 0 and 100. Higher scores indicate better quality of life.

Glycemic outcomes included HbA1c assessed by an agglutination inhibition immunoassay (DCA Vantage, Siemens Medical). IsCGM and rtCGM derived metrics included the percentage of time CGM is active, mean glucose level (mmol/L), time in range (TIR): % of readings and time 3.9–10.0 mmol/L, time above range (TAR): % of readings above 10.1 mmol/L and time below range (TBR): % of readings below 3.9 mmol/L obtained from CGM data downloads of a 14-day period prior to the study visit. These metrics are clinical targets recommended by the International Consensus on Time in Range (24).

### Statistical analysis

Data were analyzed using the open-source software R^©^ (version 4.0.3 (2020–10–10), www.r-project.org). Given the exploratory nature of the cross-sectional, observational study, no a-priori power calculation was conducted. Questionnaire scores of parent and child dyads with a normally distributed difference were compared using a paired t-test. Subgroup analyses were conducted for diabetes-technology related parameters using the Wilcoxon rank sum test: regimen was defined as using multiple daily injections or continuous subcutaneous insulin infusion users, glucose monitoring device (rtCGM or isCGM users), glucose monitoring devices without (SMBG and isCGM without alert) or with alert function (isCGM with alerts and rtCGM) and age subgroups defined as children aged below 13 years and adoelscents aged 13 years and older. Furthermore, correlations were calculated using the Spearman correlation coefficient to identify relationships between FOH and quality of life and age, metabolic control of the children, diabetes duration and CGM derived metrics and diabetes-technology related factors such as number of scans in isCGM users. P-values <0.05 were considered statistically significant.

## Results

### Study population

Fifty-nine children and adolescents older than eight years and 49 parents completed the questionnaires. Forty-four of these were parent/child dyads. Demographic data and diabetes-related parameters of both cohorts are displayed in **Table 1**. Forty-three (87.8%) of the 49 parents who completed questionnaires were mothers, six (12.2%) were fathers. The majority of the study participants were using glucose sensors as glucose monitoring devices, either rtCGM or isCGM. Mean (SD) glucose level was 9.9 (± 1.9), mean TIR was 48.9 ± 15.4%, TBR was 5.5 (± 5.2) % and TAR was 45.5 (± 17.7) %., mean percentage of time CGM was active was 83.1 (± 19.0) % during a period of 14 days. Only seven children were using self-monitoring of blood glucose with finger pricks. Devices with alerts upon low and/or high glucose levels (rtCGM and isCGM with alerts) were used by 25 of the responding children and adolescents and by 20 children and adolescents of responding parents. The use of automated insulin delivery system was low at the time of the study conduct, two participants were using a hybrid-closed loop system and five were using a predictive low glucose suspend system.

**Table 1:** Demographic and diabetes specific characteristics of the study participants.

Characteristic	Children		Children of responding parents	
	N=59		N=49	
	*Mean, SD*	*n*	*Mean, SD*	*n*
Age, years	15.1 (3.0)		12.5 (3.3)	
< 13 years		18		27
< 8 years		–		5
Gender = male		29		30
Duration of diabetes (years)	7.6 (4.1)		6.1 (3.8)	
HbA1c, current	7.6 (1.1)		7.2 (0.8)	
Regimen = CSII (%) (otherwise: MDI)		28		23
Glucose monitoring device: rtCGM/isCGM/SMBG		16/36/7		12/33/4

### Questionnaires’ data: children and parent scores

Mean child/adolescent and parent questionnaire scores are shown in **Table 2**. Parent total HFS scores were higher than the child scores indicating greater fear; 37.9 (± 14.6) vs. 32.2 (± 11.9) in children, p = 0.047. Similarly, the HFS Worry subscore was higher in parents compared to children, 17.3 (± 9.9) versus 13.7 (± 8.9), p = 0.011. No difference was observed in the Behavior subscore; the parent subscore was 20.6 (± 6.5) vs. 18.5 (± 5.7) in children, p = 0.628. There was no significant difference in general quality of life, mean (SD) child score was 84.4 (± 11.4) vs. parent score 82.5 (± 12.1), p = 0.410. However, diabetes-specific quality of life scores were lower in parents compared to children, 77.5 (± 12.7) vs. 80.8 (± 11.2), p = 0.022.

**Table 2:** Questionnaire scores of children and adolescents and their parents.

Questionnaire	Child score mean (SD)	Parent score mean (SD)	Mean difference	95% CI	P-value*
**HFS total score**	32.2 (11.9)	37.9 (14.6)	5.6	0.08 to 11.2	0.047*
Behavior subscore	18.51 (5.7)	20.63 (6.5)	0.6	-1.9 to 3.1	0.628
Worry subscore	13.73 (8.9)	17.29 (9.9)	5.0	1.2 to 8.8	0.011*
**PedsQL Diabetes total score**	80.8 (11.2)	77.52 (12.7)	- 3.873874	- 7.2 to - 0.6	0.022*
**PedsQL Generic core total score**	84.4 (11.4) [59]	82.48 (12.1)	-1.032072	-3.5 to 1.5	0.410
**PedsQL Family Impact total score**	–	79.0 (15.8)	–	–	**-**
Family function subscore	–	76.0 (20.5)	–	–	–
Health-related quality of life subscore	–	80.6 (16.1)		–	–

Data are expressed as mean scores and SD. Mean difference and p-values are derived from paired t-tests where paired questionnaire data were available, n=44. *P< 0.05 was considered significant. CI, Confidence Interval, HFS, Hypoglycemia Fear Survey; PedsQL, Pediatric Quality of Life.

### Correlations with age and metabolic control

**Table 3** shows correlations between questionnaire scores and age and metabolic control. Child diabetes specific QoL was inversely correlated with HbA1c (r = -0.339, p = 0.009). Parent reported generic quality of life and family related quality of life, but not diabetes-specific, correlated with age, (r = 0.392, p = 0.005 and r = 0.558, p<0.001) but not with parameters of glycemic control. No correlation with CGM derived metrics and diabetes duration was observed. In view of the correlation with age, HFS and QoL scores were compared between children below 13 years of age and aged 13 and older (= adolescents). Parental and child HFS scores did not differ significantly, however parents of children perceived their child’s generic and diabetes-specific quality of life to be significantly lower than parents of adolescents, **Supplemental Table 1**. In addition, parent quality of life rated on the family impact module was lower in parents of young children compared to parents of adolescents. 

**Table 3:** Spearman correlation coefficient between age, metabolic control and questionnaire scores, *p< 0.05, **p< 0.01, ***p< 0.001. HFS, Hypoglycemia Fear Survey; PedsQL, Pediatric Quality of Life.

	age	HbA1c
**HFS total score child**	0.075	0.0384
** parent**	-0.088	-0.194
**PedsQL Diabetes total score** ** child**	-0.109	-0.339**
** parent**	0.263	-0.043
**PedsQL Generic total score** ** child**	0.1102	-0.191
** parent**	0.392**	-0.018
**PedsQL Family Impact total score**	0.558***	0.142

### Impact of diabetes technology

#### Regimen and glucose monitoring devices

There was no difference in median HFS, generic and diabetes specific quality of life scores comparing child and parent questionnaire scores between rtCGM and isCGM users and between glucose monitoring devices with alerts (rtCGM and isCGM with alarm option) and without alerts (isCGM without alarm option and SMBG), **Table 4**. Comparison to SMBG users alone was not performed due to the small numbers in this group (n = 7 in children and n = 4 in children where parents completed the questionnaires). No difference was observed comparing MDI and CSII users, **Supplemental Table 2**.

**Table 4:** Questionnaire scores of real time continuous glucose monitoring (rtCGM) users vs. intermittently scanned CGM users (isCGM) and glucose monitoring devices without (self-monitored blood glucose (SMBG) and isCGM without alerts) vs. with (rtCGM and isCGM with alerts) in children and parents: data are expressed as median (IQR).

Questionnaire scores	Children: median (IQR)			Parents: median (IQR)		
	**rtCGM, n=16**	**isCGM, n=36**	**p**	**rtCGM, n=12**	**isCGM, n=33**	**p**
**HFS total score**	32 (26.5 to 39.0)	32 (23.0 to 40.0)	1.000	45 (34.0 to 54.0)	39 (26.0 to 49.0)	0.177
**PedsQL diabetes total score**	78.0 (72.6 to 89.1)	83.5 (75.3 to 92.0)	0.402	76.8 (68.1 to 81.4)	77.7 (73.0 to 84.2)	0.449
**PedsQL generic total score**	87.2 (73.1 to 92.6)	87.6 (83.6 to 94.0)	0.301	78.4 (72.1 to 86.5)	82.0 (76.9 to 95.0)	0.411
**PedsQL family impact total score**	–	–		68.9 (67.0 to 89.4)	81.5 (69.6 to 94.1)	0.238
	**Without alerts, n=34**	**With alerts, n=25**	**p**	**Without alerts, n=29**	**With alerts, n=20**	**p**
**HFS total score**	29.0 (22.0 to 37.0)	32 (27.0 to 36.0)	0.388	34 (22.0 to 49.0)	42.0 (34 to 50.0)	0.682
**PedsQL diabetes score**	82.6 (75.3 to 89.7)	80.2 (74.2 to 90.1)	0.790	82.0 (75.8 to 89.7)	76.1 (68.1 to 81.4)	0.055
**PedsQL generic score**	85.1 (81.4 to 88.8)	88.0 (74.8 to 93.4)	0.911	82.6 (71.2 to 91.7)	81.0 (72.1 to 90.0)	0.605
**PedsQL family impact total score**	–	–		82.6 (71.2 to 93.8)	79.1 (66.6 to 92.2)	0.555

P-values are derived from Wilcoxon signed rank-tests. HFS, Hypoglycemia Fear Survey; PedsQL, Pediatric Quality of Life.

#### IsCGM scanning frequency

In isCGM users, median (IQR) scanning frequency was 6.5 (5 to 10.3) scans per day in children who completed the questionnaires and 9 (6 to 16) scans per day in children of parents who completed the questionnaires. Parent total HFS score was positively correlated with the number of scans performed per day (r = 0.399, p = 0.021), parent reported diabetes-specific, generic quality of life and family quality of life was negatively correlated with the number of scans performed per day (r = -0.464, p = 0.007; r = -0.40, p =0.002, r = -0.424, p = 0.014 respectively). No significant correlation was observed between scanning frequency and child questionnaire scores. However, scanning frequency was inversely correlated with HbA1c, r = -0.353, p = 0.017.

## Discussion

This study showed that parents are more likely to experience higher levels of psychosocial burden related to their child’s diabetes than children and adolescents with T1D. This study explores psychosocial outcomes as a primary focus in the era of currently available sensors. In literature, studies are often investigating psychosocial outcomes as a secondary outcome and earlier glucose monitoring devices were used. It highlights, that psychosocial burden such as fear of hypoglycemia and impaired quality of life is still present despite continuous technological advances. A way to address and reduce this psychosocial burden may be to implement regular screening for fear of hypoglycemia and for example diabetes distress at annual controls to identify children with T1D and their families at risk and refer them to an accompanying psychological team.

In this study, specifically in the HFS Worry subscore, parents indicated a significantly higher FOH than children. It is important not to neglect parents’ FOH and to counteract it by providing parents with adequate hypoglycemia prevention and treatment resources. An option is to offer real-time remote monitoring including individual alarms of their children’s glucose levels *via* isCGM or rtCGM applications, which has been shown to reduce FOH (16). The use of remote monitoring in this study was however not documented.

Furthermore, parent-reported diabetes-specific quality of life was significantly lower than child-reported. An analysis from the SEARCH for Diabetes in youth investigating discrepancies in parent-proxy and youth reported quality of life also showed that quality of life perceptions between caregivers and youth differ significantly (25). This is in line with a more recent study showing significantly different quality of life between children and parents (26). Johnson et al. found in their cross-sectional study with 325 children and their parents, that children and parents with the greatest FOH reported a significantly reduced quality of life (9). However, in this study parents and children were not compared directly to each other.

Compared to other studies investigating FOH and QoL in an experimental trial setting before and after starting glucose sensors, children and parents scores of our study are in range comparable to scores obtained after rtCGM initiation at the end of the respective study period (16, 18, 27, 28). HFS scores before rtCGM start appeared to be higher than in our study. However, caution should be applied when comparing scores across different studies due to different study populations, trial settings and device availability. QoL life scores are situated in the comparable ranges (28).

In our study, child diabetes-specific quality of life scores were inversely correlated with HbA1c. In literature, some studies report a similar association (9, 29), whereas others did not find such a correlation (25). However, causality between psychosocial and glycemic outcomes should not be automatically assumed due to individual, multifactorial components of diabetes management. Parent-reported generic and diabetes-specific quality of life was not associated with HbA1c, suggesting that parent-reported quality of life may be more determined by diabetes management behaviors and not glycemic control (30). Parent-reported generic quality of life correlated with the child age, i.e. parents of younger children reported lower generic quality of life. Young children are less independent and therefore their parents are and may feel more responsible regarding their child’s diabetes management. Therefore, these parents might experience a higher psychosocial burden. Furthermore, particularly nocturnal diabetes management requires waking up during the night to measure the child’s glucose level via isCGM or finger-pricking and could explain the perceived poorer quality of life in younger children. Parent and child FOH did not correlate with age and HbA1c, in line with a study investigating well-being of parents with very young children (30). This suggests that glycemic control is not the main driver of FOH in parents of children with type 1 diabetes.

No differences in HFS scores and quality of life were observed between rtCGM vs. isCGM users, users of glucose monitoring device without vs. with alerts and insulin pump vs. MDI users. Due to the cross-sectional design of the study, no longitudinal data of the FOH and QoL levels of the study participants prior to switch to a specific regimen or glucose monitoring device was available.

However, parents of children who scanned their isCGM frequently or where the parents scanned the sensor themselves reported significantly higher FOH. Potentially, parents with higher FOH scan more frequently to check for and prevent hypoglycemia. When scanning more frequently they may consequently have a higher probability of counteracting and preventing hypoglycemia by adjusting diabetes management decisions. Moreover, higher scanning frequency correlated with lower diabetes-specific, generic quality of life and family quality of life. Higher FOH in parents, as described above, may lead to decreased quality of life (9). Similar to our study, Urakami et al. showed that higher scanning frequency (> 12 times/day) can be associated with better glycemic control (17, 31). In this study frequent scanning has been shown to lead to a reduced frequency of hypoglycemia (17).

A strength of this study is to evaluate psychosocial outcomes in children and adolescents and their families using currently available glucose sensors in real-life. Furthermore, comparison of child and parent scores may help to identify problems of different target groups and provide more patient-centered education. Such interventions may include for example interventions delivered by telehealth such as a recently trialed video-based intervention to reduce FOH in parents of young children with T1D that seems to improve fear of hypoglycemia in parents after the intervention (32). Measures such as the HFS could be performed at the annual control at the same time screening for associated autoimmune disorders and long-term complications occur. Families at risk could be identified systematically and referred to the psychological team to receive a more in-depth assessment and more personalized treatment and education. This study has however some limitations. The cross-sectional study design does not allow for comparisons over time and establishing causalities, for instance before and after the start of using a rtCGM or isCGM. Furthermore, the relatively small sample size does not allow to generalize the results. Also, only a small number of participants were using a hybrid-closed loop system when the study was conducted – real-life uptake of these systems has increased markedly over the past year and it will most likely also influence FOH and quality of life as already demonstrated in a large multicenter randomized controlled trial (33). More and longitudinal studies comparing psychosocial outcomes before and after a certain duration of rtCGM/isCGM use are needed with the ultimate aim to identify further factors influencing patient reported outcomes in relation to diabetes technology use.

To conclude, this study showed that psychosocial burden such as FOH and impaired quality of life is present specifically in parents of young children with T1D using currently available glucose monitoring devices and children and their parents often perceive this burden differently. This highlights the need for identification of families at risk for high psychosocial burden and for family-based education and treatment resources in addition to rapidly advancing diabetes technology to support parents in diabetes management, provide more optimized family centered-care and offer enhanced technological solutions.

## Data Availability Statement

The raw data supporting the conclusions of this article will be made available by the authors, upon reasonable request.

## Ethics Statement

The studies involving human participants were reviewed and approved by Ethikkommission Nordwest- und Zentralschweiz. Written informed consent to participate in this study was provided by the participants’ legal guardian/next of kin.

## Author Contributions

VG contributed to the data collection, data analysis and manuscript preparation and reviewed and edited the manuscript. SB and MH contributed to participant recruitment and reviewed and edited the manuscript. GS contributed to the study design and reviewed and edited the manuscript. M-AB was responsible for the study design, data collection, data analysis and manuscript preparation. All authors approved the final version of this article.

## Funding

M-AB was funded by a research fellowship provided by the Research Board of the University Children’s Hospital, Basel, Switzerland. The study was supported by a grant of the Diabetes Foundation Switzerland (Schweizerische Diabetes-Stiftung).

## Acknowledgments

We thank all children and adolescents and their families who participated in the study. We also thank Kris Denhaerynk for his statistical support.

## Conflict of Interest

The authors declare that the research was conducted in the absence of any commercial or financial relationships that could be construed as a potential conflict of interest.

## Publisher’s Note

All claims expressed in this article are solely those of the authors and do not necessarily represent those of their affiliated organizations, or those of the publisher, the editors and the reviewers. Any product that may be evaluated in this article, or claim that may be made by its manufacturer, is not guaranteed or endorsed by the publisher.
