# Supplementary material for: Fear of hypoglycemia and quality of life in young people with type 1 diabetes and their parents in the era of sensor glucose monitoring
Source: Front Endocrinol (Lausanne). 2022 Jul 28;13:958671. doi: 10.3389/fendo.2022.958671 (PMC9367634; doi:10.3389/fendo.2022.958671)
Supplement: Supplementary file 1 [file DataSheet_1.docx]

Supplementary documentation

Supplemental Figure 1: Study design.


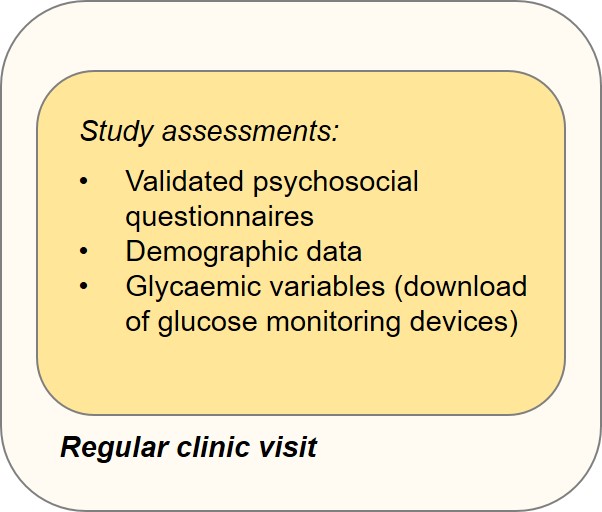


Supplemental Table 1: Questionnaire Scores of children *vs* adolescents

#### Children < 13 years of age vs. adolescents ≥ 13 years of age in children and parents: data are expressed as median (IQR), p-values are derived from Wilcoxon rank sum tests. HFS, Hypoglycemia Fear Survey; PedsQL, Pediatric Quality of Life.

| **Questionnaire scores** | **Children: median (IQR)** | |  | **Parents: median (IQR)** | |  |
| --- | --- | --- | --- | --- | --- | --- |
|  | **< 13 years** | **≥ 13 years** | **p** | **< 13 years** | **≥ 13 years** | **p** |
| **HFS total score** | 28.0 (21.5 to 36.3) | 32.0 (24.5 to 40.0) | 0.204 | 40.0 (29.3 to 49.8) | 34.0 (24.0 to 49.0) | 0.323 |
| **PedsQL diabetes total score** | 82.1 (74.1 to 92.2) | 82.0 (74.6 to 89.7) | 0.966 | 77.0 (69.2 - 82.3) | 82.4 (75.8 - 91.4) | 0.031 |
| **PedsQL generic total score** | 82.5 (75.8 to 88.8) | 88.0 (83.3 to 93.1) | 0.098 | 77.5 (71.8 - 83.1) | 89.2 (81.4 - 98.8) | < 0.001 |
| **PedsQL family impact total score** | - | - |  | 70.0 (63.1 to 86.4) | 93.5 (79.1 to 96.4) | < 0.001 |

Supplemental Table 2: Questionnaire Scores of MDI vs CSII Users

#### Multiple daily injections (MDI) users vs. continuous subcutaneous insulin infusion (CSII) users in children and parents: data are expressed as median (IQR), p-values are derived from Wilcoxon rank sum tests. HFS, Hypoglycemia Fear Survey; PedsQL, Pediatric Quality of Life.

| **Questionnaire scores** | **Children: median (IQR)** | |  | **Parents: median (IQR)** | |  |
| --- | --- | --- | --- | --- | --- | --- |
|  | **MDI , n=31** | **CSII, n=28** | **p** | **MDI, n=26** | **CSII, n=23** | **p** |
| **HFS total score** | 28.0 (22.0 to 37.0) | 33.0 (26.0 to 41.0) | 0.215 | 35.0 (26.0 to 48.0) | 40.0 (34.0 to 50.0) | 0.231 |
| **PedsQL diabetes total score** | 83.1 (71.8 to 90.3) | 80.8 (76.0 to 89.5) | 0.994 | 81.9 (74.6 to 89.7) | 77.3 (68.8 to 82.4) | 0.259 |
| **PedsQL generic total score** | 83.9 (78.3 to 90.9) | 87.8 (80.1 to 92.6) | 0.634 | 83.1 (76.9 to 93.8) | 79.8 (72.2 to 88.1) | 0.336 |
| **PedsQL family impact total score** | - | - |  | 81.2 (69.6 to 91.3) | 79.6 (67 to 95.0) | 0.926 |
